# “Mummy, Can I Join a Sports Club?” A Qualitative Study on the Impact of Health-Promoting Schools on Health Behaviours in the Home Setting

**DOI:** 10.3390/ijerph182212219

**Published:** 2021-11-21

**Authors:** Marla T. H. Hahnraths, Maartje Willeboordse, Annick D. H. M. Jungbauer, Corina de Gier, Carlien Schouten, Constant P. van Schayck

**Affiliations:** Department of Family Medicine, Care and Public Health Research Institute (CAPHRI), Maastricht University, 6200 MD Maastricht, The Netherlands; m.willeboordse@mulierinstituut.nl (M.W.); annick.jungbauer@hotmail.com (A.D.H.M.J.); corinadegier@gmail.com (C.d.G.); carlien.schouten@outlook.com (C.S.); onno.vanschayck@maastrichtuniversity.nl (C.P.v.S.)

**Keywords:** primary school, health-promoting school, qualitative research, physical activity, nutrition, behavioural change

## Abstract

Information regarding school-based health-promoting interventions’ potential effects in the home environment is scarce. Gaining more insight into this is vital to optimise interventions’ potential. The Healthy Primary School of the Future (HPSF) is a Dutch initiative aiming to improve children’s health and well-being by providing daily physical activity sessions and healthy school lunches. This qualitative study examines if and how HPSF influenced children’s and parents’ physical activity and dietary behaviours at home. In 2018–2019, 27 semi-structured interviews were conducted with parents from two HPSFs. Interviews were recorded and transcribed, and data were coded and interpreted through thematic analysis. HPSF resulted in various behavioural changes at home, initiated by both children and parents. Parents reported improvements in healthy behaviours, as well as compensatory, unhealthy behaviours. Reasons for behavioural change included increased awareness, perceived support to adopt healthy behaviours, and children asking for the same healthy products at home. Barriers to change included no perceived necessity for change, lack of HPSF-related information provision, and time and financial constraints. Both child-to-adult intergenerational learning and parent-initiated changes play an important role in the transfer of health behaviours from school to home and are therefore key mechanisms to maximise school-based health-promoting interventions’ impact.

## 1. Introduction

Childhood is an important period in life for the development of lifestyle habits (e.g., physical activity (PA) and dietary habits), many of which are known to persist throughout adult life [1]. Unhealthy habits during childhood increase the risk of developing overweight and obesity, which has immediate negative consequences for children’s physical, mental and social health. Furthermore, childhood overweight and obesity is often maintained during adult life and increases the risk of non-communicable diseases such as coronary heart disease and type 2 diabetes [2]. Considering this, the formation of healthy lifestyle habits early in life is vital to obtain long-lasting health benefits.

The ecological systems theory (ETS) states that behaviour is influenced by the various ecological and social contexts in which a person resides [3,4,5,6]. Children spend a considerable amount of time in the home and school environment, making them both important settings when it comes to influencing behaviour. According to the ETS, the various environments not only affect behaviour directly but also influence each other [3,4,5,6]. This means that lifestyle habits learned at home influence children’s behaviour at school, and vice versa. As a result, school-based interventions promoting healthy lifestyle habits can also influence children’s behaviours at home. The healthy habits learned at school can be transferred to the home context via children, leading to healthy behavioural changes at home. Alternatively, children (or parents) might feel that the healthy changes at school can compensate unhealthy behaviours at home, resulting in a justification of unhealthy changes in the home context [7].

Over the years, many school-based health-promoting interventions have been developed and implemented. Despite various effect evaluations, the impact these school-based interventions can have on health behaviour in the home context is seldom specifically investigated [8,9]. Gaining more insight into how changes in the school context might affect the home setting is therefore vital to optimise the potential of lifestyle interventions targeting children.

The Healthy Primary School of the Future (HPSF) is a Dutch initiative that aimed to improve the health and well-being of primary school-aged children by sustainably integrating health promotion within the whole school system [10]. As part of a comprehensive research project, two “full HPSFs” implemented a daily healthy school lunch and structured PA sessions during the extended lunch break time [10]. Earlier reports of this study revealed favourable effects of HPSF on children’s BMI z-score, waist circumference, and PA and dietary behaviours (e.g., school water consumption and vegetable and dairy intake during lunch) [11,12]. Additional quantitative analyses revealed no statistically significant favourable or adverse intervention effects on children’s PA or dietary behaviours in the home context [13]. However, in informal conversations with several stakeholders (e.g., teachers and parents), various behavioural changes in the home setting were mentioned. Possibly, the HPSF-related changes at home were more extensive than could be captured using quantitative research methods, which makes additional qualitative research desirable. The present study therefore aims to identify and further investigate if there are patterns of behaviour change in the home environment that resulted from exposure to the HPSF intervention. For this purpose, qualitative research methods are used to answer the following research question: “Does the Healthy Primary School of the Future lead to changes in health behaviours (especially physical activity and dietary behaviours) of children and parents in the home environment, and if so, what are the processes behind these changes?”

## 2. Materials and Methods

### 2.1. Setting and Study Design

The present study is part of a comprehensive quasi-experimental research project that investigates the effects of HPSF. For this purpose, data were gathered in eight primary schools (two full intervention schools (full HPSFs), two partial intervention schools (partial HPSFs), and four control schools) on various outcomes such as children’s PA and dietary behaviours and anthropometrics. The current study consists of a qualitative analysis focussed on the two full HPSFs. Aim is to investigate if HPSF resulted in changes in PA and/or dietary behaviours of children and parents in the home environment and to further understand the processes behind any changes that might have occurred. For this purpose, semi-structured interviews were conducted with parents/caregivers of pupils from the two full HPSFs. The need for ethical approval for the overall study was waived by the Medical Ethics Committee Zuyderland in Heerlen (14N-142). Additionally, permission was given by the Medical Ethics Committee Zuyderland to actively approach all parents/caregivers of pupils from the two full HPSFs for participation in an interview, including parents who did not sign an informed consent for their child(ren) to participate in the overall HPSF study. This was performed to minimise selection bias, as parents already participating in the overall research project might have had a more positive opinion regarding HPSF, possibly resulting in an incomplete representation of the situation. In accordance with the Medical Research Human Subjects Act, parents who did not yet give permission for participation in the overall research project were asked to fill in an informed consent before participating in an interview.

### 2.2. The Healthy Primary School of the Future

HPSF is an innovative whole-school approach to health. Aim of the project was to improve children’s health, lifestyle, and overall well-being via an innovative school day [10]. Implementation of HPSF started in the fall of 2015 in various primary schools in Parkstad, a region in the southern part of the Netherlands. This region is characterised by a low social economic status (SES) and substantially higher obesity rates than the national average [14,15]. Parents were actively involved in the development of HPSF, and a parental vote took place in all participating schools prior to implementation of HPSF.

Four control schools and four intervention schools (two full HPSFs and two partial HPSFs) were enrolled in the HPSF research project [10]. The two full HPSFs implemented changes in both nutrition and PA by offering a free healthy school lunch each day, combined with daily structured PA and cultural activities of at least 60 min during the prolonged lunch break [10]. This prolonged lunch break was realised through extending the regular school hours with on average 30 min. All changes were supervised by pedagogical staff. All intervention components were child oriented, and there were no components targeting parents. Parents were informed regularly about the intervention at school through newsletters and occasional information sessions. In addition, they were asked to volunteer during the various activities at school. A detailed description of study design, intervention, and recruitment is described elsewhere [10].

### 2.3. Participants

Interview participants were recruited between November 2018 and February 2019 among parents/caregivers of children who were enrolled in one of the two full HPSFs (*n* = 677 children; corresponding to ±509 families). Parents/caregivers were excluded from participation if they themselves or their partner worked as a teacher or pedagogical staff member at one of the full HPSFs.

**Figure 1:**
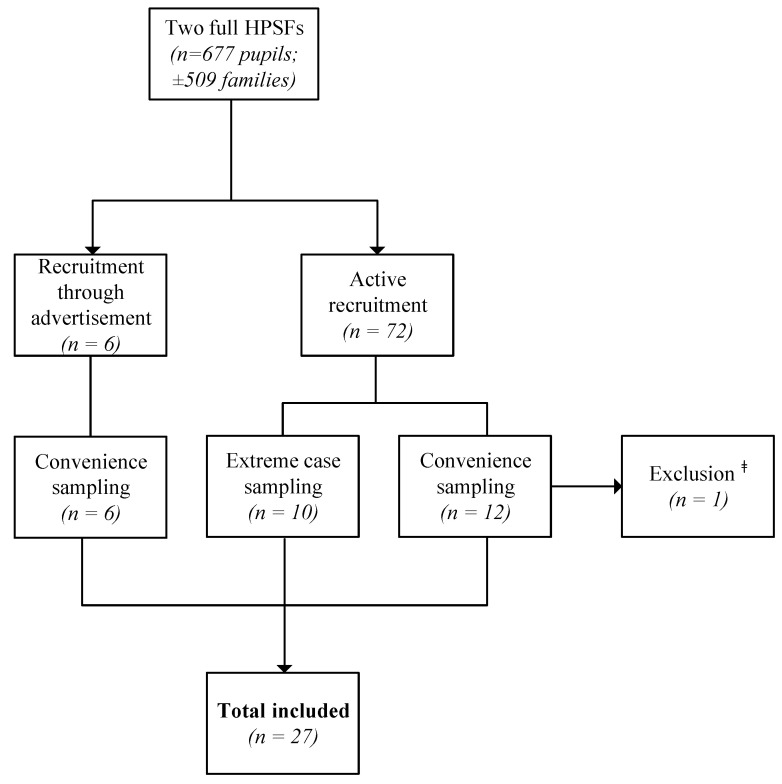
Flow chart of participant recruitment. ^ǂ^ Excluded because the participant was a pedagogical staff member at one of the full HPSFs. Abbreviations: HPSFs, Healthy Primary Schools of the Future.

Participants were recruited using an advertisement in the school’s (digital) newspaper and via active recruitment by researchers in the schoolyard after school hours and during two school events. Parents/caregivers were asked to participate in an interview to give their opinion about HPSF. The underlying research question (i.e., investigating any HPSF-related processes of change in the home environment) was not explained during recruitment. After initial recruitment, two different sampling strategies were applied. First, convenience sampling was applied, where participants were selected based on their availability and willingness to participate, resulting in the inclusion of eighteen participants. Second, extreme case sampling was applied to increase heterogeneity and to reach data saturation. Through this method, ten additional participants were included (Figure 1). Extreme cases were those parents/caregivers with an extreme or divergent opinion regarding HPSF, both positive and negative. To select extreme cases among the actively recruited parents/caregivers, school project leaders served as an important information source as they had a good overview of all extreme cases within their school.

### 2.4. Interviews

Three researchers involved in the HPSF research project (R.v.G., male; C.d.G., female; and C.S., female) carried out the interviews using a semi-structured interview guide (Supplementary File S1). This interview guide was developed in consultation with various stakeholders (e.g., parents, teachers, and pedagogical staff members). Using their observations and experiences with HPSF, various important themes (e.g., HPSF appreciation, influences on PA, and dietary behaviours) were identified and incorporated in the guide, which was subsequently pilot tested on parents among university staff. Open and nonsuggestive questions were used to prevent socially desirable answers and to ensure participants were not steered in a certain direction by question formulation. During the process of data collection, the interview guide was continuously revised and adapted, based on temporary analyses. In addition to the interview guide, interviewers used additional questions and follow-ups in response to the issues discussed during the interviews.

In consultation with the participants, a date and place for the interview was selected. Most interviews were conducted at home, although some participants preferred to be interviewed at school or at the university. Before the interview started, the interviewer briefly introduced him-/herself, and participants were verbally encouraged to express their true opinion, both positive and negative. After each interview, the interviewers reflected on the atmosphere and context, which helped to understand the family dynamics during the coding and analysis process.

### 2.5. Data Analysis

As there is little theory available regarding behavioural changes in the home context resulting from school-based interventions, data analysis followed a deductive grounded theory approach [16]. Interviews were recorded using a digital voice recorder and transcribed verbatim. Transcripts were not returned to participants for comments and/or corrections. After transcribing, NVivo 12 software (QSR International Pty Ltd., Doncaster, Australia) was used to structure, analyse, and interpret the interviews. Through thematic analysis, a structured framework of nodes was developed and tested by three researchers (A.D.H.M.J., C.S., and M.W.) using two rich interviews. One researcher (A.D.H.M.J.) systematically coded the remaining interviews by placing corresponding quotes under the different nodes in the framework, revising the framework when necessary. The coding process, which mainly involved open and axial coding, was continuously documented using memos. Ambiguities were discussed with two other researchers (C.S. and M.W.), and annotations were kept regarding socially desirable answers to reflect on the researcher’s objectivity. Answers that were perceived as socially desirable were included but were considered less significant than answers that were perceived as more sincere. The final coding framework consisted of various nodes (e.g., health behaviours in the home context and at school, participants’ opinion regarding HPSF, and factors of influence on health behaviours at home). Data saturation was defined by three pillars: (1) there are enough data to replicate the study, (2) the ability to obtain new data is reached, and (3) further coding is no longer practicable [17]. After coding all interviews, a structured overview of the data was generated [18]. Each node was then individually analysed, and themes were distracted to answer the research question. Interview participants did not provide feedback on the findings.

## 3. Results

### 3.1. Background

In total, 28 in-depth interviews involving 32 parents/caregivers were conducted between December 2018 and March 2019 before data saturation was reached. One interview was excluded because after the interview it became clear that the participant was a pedagogical staff member at one of the full HPSFs (Figure 1). The average duration of the interviews was 47 min, with a minimum duration of 23 min and a maximum of 70 min. Of the 27 interviews included, 16 were with parents/caregivers of children from HPSF1, and the remaining 11 involved parents/caregivers of children from HPSF2. Participants were predominantly positive about the HPSF concept. In 21 of the 27 (77.8%) interviews, participants indicated they voted in favour of HPSF implementation at the start of the project or took it into account when choosing a primary school for their children (Table 1). This was in the same order of magnitude as the percentage of the total parental population that voted in favour of HPSF before the implementation started in 2016 (86% in HPSF1 and 91% in HPSF2). Despite the overall positive opinion regarding HPSF, participants were critical about the execution of the project, in particular concerning food waste, communication, and supervision. Participants reported that their children were generally positive about HPSF; they liked the communal lunch and exercising together.

### 3.2. Processes of Change

Participants were not always aware of changes in their own and/or their children’s behaviour following the start of HPSF. Most participants reported various behavioural changes in the home setting since the start of HPSF, both healthy and unhealthy. The interviews revealed two main processes behind behavioural changes in the home environment. First, children played a central role in transferring the intervention’s content from school to the home environment, influencing the rest of their families. Furthermore, parents were directly influenced by HPSF and subsequently initiated behavioural changes in the home context themselves. In short, HPSF had a direct influence on the behaviour of both children and parents, and children and parents influenced each other’s behaviours.

#### 3.2.1. Children as Change Agents

Children had an influential role in the transfer of HPSF’s contents to the home environment. At school, children were exposed to new food products and engaged in different forms of PA. Children reacted to this by initiating both healthy and unhealthy changes in dietary and PA behaviours at home. Many participants reported that their children asked for healthy products that they had come to know in school, recognised them in the supermarket, or asked questions about their family’s dietary behaviours, *“Yes, I think, she [daughter] is actually quite aware of it. Because when I grab something that is less healthy, she says: “Mum that is not really healthy”. So she is really aware of that. So it is kind of in her system already”.* (HPSF2.10). Children not only introduced new products in the home environment, they also transferred dietary behaviours learned at school to the home setting. Several participants reported that their children tasted new products more easily or learned to drink water as their main source of fluid, *“It was pretty quickly that we noticed it at home when we introduced a new food product. Especially during the evening meal or with new sandwich spreads, that they [the children] were less hesitant, less disgusted, less: “I do not want to taste that”. They tasted it and they just ate it. You notice that very quickly”.* (HPSF2.9). On the other hand, children also used the fact that they ate healthily and exercised at school as an excuse to compensate for these behaviours at home. *“…Like, “Mum, we can have some candy because at school we ate healthily the whole day!” You know…”* (HPSF1.6).

The extent to which parents reacted to their children’s behavioural changes varied across participants and could lead to healthy and unhealthy changes in the home environment. When children asked for new food items or showed new dietary behaviours, some parents supported this by buying the requested items and serving new products. Often, parents were motivated by their children’s enthusiasm and tried the new products as well, *“When we walked through the supermarket for groceries, for the weekend or something, for the week. And then she [daughter] said: “Oh we have those crackers at school too!”.... Yeah, so actually our entire family was included. Not just the children, but because they were so enthusiastic and recognised things from what they got at school. If they showed that and I thought… Yes, then we would try that”.* (HPSF1.6). Participants did not report situations in which their children showed healthy behavioural changes which they did not want to facilitate. However, most participants did indicate that it was not always possible to provide healthier products or more opportunities to be physically active, for example due to practical and financial constraints. Some participants mentioned that the price and shelf life of food products played an important role in their consideration to buy new products. If the requested food products had a short shelf life and/or children did not eat them at home causing food waste, participants were less likely to buy these items again. Additionally, one participant said her children asked for blueberries, which she could not afford. Alternatively, she offered her children less expensive fruit items. For PA, logistics were often a limiting factor. One participant mentioned that her daughter would love to join a sports club, but because all sport activities were in the evening and there was only one bus drive at night, she could not let her daughter join, *“She [daughter] would love to join a sport. But it is not possible for me to arrange that, because everything is during the evening, around 6–6:30 pm. And yeah, by the time I get home it is 9–9:30 pm. So, that is not possible on a school night”.* (HPSF2.10).

Some participants facilitated unhealthy behaviour through allowing their children to eat more snacks, drink soft drinks, or have more screen time at home than before HPSF. Most participants justified this behaviour by stating that it is important to have a balance between healthy and unhealthy behaviours, *“At home she [daughter] likes to drink something like juice. So it is… She can discuss about it like: “I had to drink water all day already, and now you tell me to drink water as well!” Well and then I think, when she is home… It is not that big of… She can have juice”.* (HPSF1.11).

On the other hand, there were also various participants who did not facilitate unhealthy behaviour. They did not answer to their children’s wish for unhealthy snacks or screen time, and they sometimes even stimulated healthy behaviour instead, *“When he [son] comes home, and he uses the same excuse if he wants cookies, he says: “But I already ran so much outside today. Can I not just play video games?” So that it even goes in the wrong direction. And then I say “Look, the weather is nice! There is snow; you go get your ski suit!””* (HPSF1.10).

#### 3.2.2. Parents Initiate Behavioural Change

Next to behaviour changes initiated by children, parents also introduced healthy and/or unhealthy changes in family dietary and PA behaviours.

Because the school employed certain rules surrounding the school lunches and PA activities, parents could adopt these rules and habits more easily at home. Many participants indicated that they felt stimulated to reflect on their dietary and PA behaviours and that they intentionally implemented changes, *“My husband and I talked about it. I thought the mornings were very messy, and they [the children] would eat in front of the television. It just did not feel right. So we talked about it the next morning and now we all eat at the table. Statement made. So now, eh, we have this insight”.* (HPSF1.6). These participants said they were stimulated and even felt supported to introduce new rules or maintain existing rules more strictly, *“I know that I can be indulgent, like: “You have to eat a sandwich and it cannot be a sandwich with just butter”. But why not? So I actually started thinking about it consciously. Where, for example, before I would say: “Grab a sandwich with chocolate sprinkles”. Now they [the children] just have to eat a bare sandwich. That is the same at school”.* (HPSF1.6). Other participants indicated that their children were used to these rules at school, and, therefore, when they would apply them at home, the children seemed to adjust to them more easily, *“Interviewer: And do they [the children] like it when they learn new things at school, regarding food? Mother: “Yes very much, I also notice it at home sometimes, when there is something on the table. They say: “I do not like that!” We ask them: “Did you already taste it? First taste it!” And then they say: “Oh I do like it!””* (HPSF1.3). An important reason for many parents to implement these changes in the home environment was that they wanted to be a role model for their children, *“Interviewer: Why do you think you started to be more aware of your behaviour or look at it differently? Mother: Yeah, also because you want to set an example, also for the children of the healthy primary school of course”.* (HPSF1.3). Some participants mentioned they consciously chose to enrol their child in one of the HPSFs, and they wanted to continue the school’s approach to health at home, *“The fact that it was a HPSF was a reason for us to choose this school and then I think you should not give him [son] unlimited access to candy at home. He spends many hours at school and most hours of the day, he is used to this structure. It does not work for our son to switch everything once he gets home. … It is of course nice that everything at school is healthy and it is a small effort to apply that at home as well”.* (HPSF1.2).

There were also some participants who initiated unhealthy behaviours. Most of these participants reported they felt less “guilty” about their choices regarding diet and PA. They let the fact that their children already ate healthily or exercised at school influence their decisions at home, *“I do take it into account when it comes to them [the children] joining a sports club right now, because normally I think that is really important. My daughter had swimming lessons and I thought my sons would do soccer or something. But right now, I think: “I need to have a break”. And I am very honest, at the moment with work and stuff it is very busy. At least they exercise at school. And yeah, indeed I think: “Okay, they did have their exercise today”.”* (HPSF1.1).

### 3.3. Other Causes of Change

Not only did the school lunch and PA sessions trigger changes in the home setting, the prolonged school hours also influenced health behaviours at home. Because there was less time between the end of the school day and dinner, children were less likely to snack before the evening meal. One participant reported that her children had dinner earlier with their babysitter, because otherwise, they would start snacking. Contrarily, prolonged school hours resulted in less time for after school activities (e.g., playing with friends or sports activities) and/or tired children, which negatively affected PA behaviours at home for a small number of participants, *“Interviewer: Is it possible, like you said, with the prolonged school hours, is there time for those after school activities? Mother: Yes, there is. [However] We did quit dance classes, because our child was very tired and I do notice the school days are very long. So if I could change one thing, I would say we would shorten school hours”.* (HPSF1.4).

### 3.4. Barriers to Change

Besides the reported healthy and unhealthy changes, many participants also indicated that their dietary and PA behaviours had not changed since the start of HPSF. Important barriers to change were participants’ beliefs and habits regarding their dietary and PA behaviours. Participants who did not report major changes since the start of HPSF usually indicated that change was not deemed necessary. They perceived their diets as already healthy and balanced, their children as good eaters and/or were already satisfied with the amount of exercise they and their children engaged in. Some participants perceived HPSF as complementary to their already healthy diet at home. A minority of participants indicated that they considered the intervention unnecessary and sometimes even to be meddling, *“No, we did not start eating differently at home or deal with food differently. And it is not really that we have become much more aware of it. I think we were on the right track at home, even when they [the children] were small. So I do not think: “Oh, now I have suddenly seen the light” because things are different at school or something”.* (HPSF1.16).

Several participants noted the difficulty to change their current habits, even when they were aware of their behaviours and the things they wanted to change, *“I think that you kind of have a pattern. And those patterns are hard to break. We did adjust our habits slightly because of our family situation and by doing certain little things and not doing other little things”.* (HPSF2.11). Furthermore, even if children changed their behaviour at school, it did not always automatically lead to a change at home, *“Tasting new foods, yes, they [the children] do that at school of course, but that does not mean that it is easier at home, though. At home, they are just normal children who say: “I do not like the vegetables in the macaroni”. I cannot really say that that has changed a lot because they have to taste at school. Because what is at school is at school. And at home is still just at home”.* (HPSF1.16).

As mentioned before, other barriers to change in dietary and PA behaviours at home were time management, logistics, and financial capability. Many parents worked during the day, children came home late from school, and in the evening, various sports and other activities were planned. Evening meals often had to be prepared quickly, and there was limited time for exercise, *“Look, if we both work and my sister has to pick up the children from school. Yeah, then it is often an easy meal, 20 min and it is ready to be served”.* (HPSF2.6). In addition, a few parents reported that the lack of information provision regarding HPSF’s contents at school sometimes made it difficult to implement the same changes at home, *“With the PA activities, yeah what I miss is that I do not know between which activities they [the children] can choose at school. That would be a suggestion to also share that, because then you can also initiate that conversation at home. Like for example: “Good that you always do crafts, but maybe it would also be nice to play dodgeball sometimes”. Or, “What do you find exciting about it?” That you can guide them a bit in that as well”.* (HPSF1.6).

### 3.5. Spectrum of Behavioural Change

**Figure 2:**
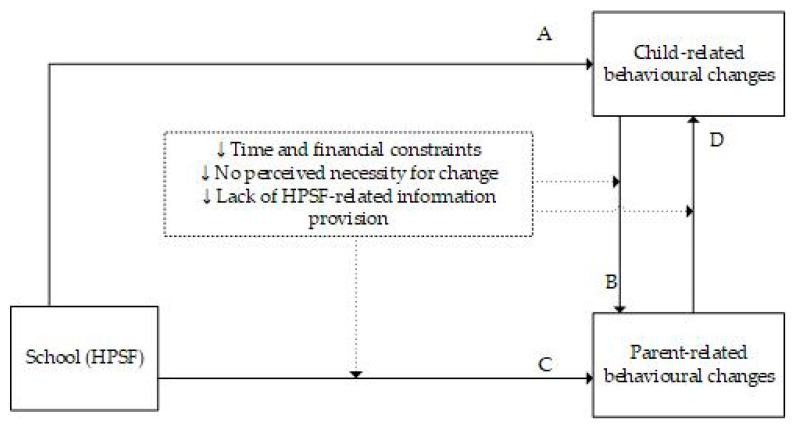
Schematic representation of the main processes behind behavioural change in the home environment. A. HPSF leads to behavioural changes in children through exposure to new food products and new forms of physical activity at school. Children react to this by, e.g., wanting the same healthy food products at home (healthy change) or by asking for snacks or more screen time (unhealthy change). B. Parents react to their children’s behavioural changes by, e.g., facilitating their children’s healthy dietary wishes and becoming enthusiastic about the new products as well (healthy changes) or by allowing their children to eat more snacks or have more screen time (unhealthy changes). C. HPSF directly leads to behavioural changes in parents. Parents feel stimulated to reflect on their dietary and PA behaviours, and they feel supported to introduce new rules or maintain existing rules more strictly. Additionally, many parents want to be a role model for their children, which is an important reason for them to implement healthy changes at home. Contrarily, parents can initiate unhealthy changes at home, as they feel that it is important to have a balance between healthy and unhealthy behaviours. D. Children react to their parents’ behavioural changes by e.g., adjusting to newly implemented dietary rules more easily (healthy change). Time and financial constraints, no perceived necessity for change, and lack of HPSF-related information provision can have a negative influence on the extent to which parents initiate behavioural changes at home following introduction of HPSF at school, indicated with the dashed arrows. Abbreviations: HPSF, Healthy Primary School of the Future.

The interviews displayed a wide spectrum in the extent to which changes in dietary and PA behaviours at school were transferred to the home context. Participants who were already satisfied with their family’s dietary and PA behaviours prior to the implementation of HPSF generally reported less change in the home setting since the start of the intervention. Other participants reported that they had become aware of their behaviour and subsequently implemented changes at home. In addition, the extent to which changes were implemented differed for dietary and PA behaviours. In general, changes in dietary behaviours were most often mentioned and discussed in more detail by participants than changes in PA behaviours. In addition, participants often reported healthy changes in dietary behaviours while they did not change or even mentioned unhealthy changes related to PA, and vice versa. This resulted in a spectrum of stages in behavioural changes that was not only observed between families but also within a family. When healthy changes occurred, participants most often reported that the child initiated these changes, for example, through asking for specific food products or wanting to join a specific sports club, *“At a certain moment he [son] mentioned basketball. I asked him: “Where did you come to know basketball?” and he said: “Yeah I did that at school”. So regarding that, he sees multiple sports”.* (HPSF1.7). In Figure 2, an overview of the main processes of behavioural change in the home environment following introduction of HPSF at school is presented.

## 4. Discussion

To maximise an intervention’s effectiveness, the acquired behavioural changes should be continued outside the controlled setting of the intervention. Insight into the processes of this transfer of intervention effects to new settings is therefore vital to improve an intervention’s impact. The present study aimed to explore the processes behind changes in dietary and PA behaviours of children and parents in the home context since the start of HPSF. The results illustrate that behavioural changes at home were initiated by both children and parents and led to healthy and unhealthy changes. Children proved to be important change agents in the transfer of HPSF’s contents to the home context. Following the intervention at school, children changed their behaviour at home, and parents responded by facilitating this to a greater or lesser extent. These findings expand on previous research suggesting that children can promote healthy dietary behaviours by influencing their parents during food shopping [19]. Parents might be more open for behavioural changes when they are proposed by their children instead of other information sources. This so-called child-to-adult intergenerational learning (IGL) has previously been described as a promising strategy to influence parents’ views and behaviours in relation to various topics (e.g., sustainability and the use of modern technology) [20,21,22,23,24]. As the present study illustrated the important role child-to-parent IGL plays in the transfer of health behaviours, this process could be stimulated more extensively in future health-promoting interventions (e.g., through homework assignments or family activities to stimulate parental involvement).

Besides the changes introduced by children, parents were also directly influenced by HPSF. As a result, they brought about change mainly by adjusting the rules regarding dietary and PA behaviours at home. Parents felt stimulated and supported to consciously reflect on their family’s behaviour and subsequently adopt new rules or uphold existing ones more strictly. Although HPSF’s primary target group was the children and the intervention did not specifically focus on parental involvement, these results suggest that the implemented changes also directly affected parents. However, several parents indicated they found it difficult to identify (un)healthy foods or could not comply with their child’s healthy requests because they were not familiar with the specific products that were served at school. In addition, various parents mentioned the lack of information provision regarding the PA activities that were organised at school, which might explain the fact that during the interviews, nutrition-related changes were discussed in more detail than PA-related changes. Better information provision regarding the different intervention components (e.g., the various food products and PA activities provided at school) might increase parental involvement and support, which could lead to an increased impact of HPSF in the home setting. Another way to increase parental involvement could be to organise activities for children and parents together (e.g., cooking workshops), which might also stimulate child-to-parent IGL as previously discussed.

Various parents reported that their children or they themselves engaged in unhealthy behaviours at home because they felt it was being compensated by the healthy changes implemented at school. These observations can be linked to the concept of compensatory health beliefs (the belief that one can compensate the negative effects of unhealthy behaviours with the positive effects of healthy behaviours) that has previously been described in relation to various health behaviours such as dietary intake and PA [7,25,26,27,28]. In the present study, no clear pattern in the occurrence of unhealthy behaviours at home could be observed. Rather, these behaviours were present across participants, were initiated by both children and parents, and occurred in relation to dietary as well as PA behaviours. These observations indicate that the implementation of HPSF at school might have led to a new decisional process at home when it comes to PA and dietary behaviours. Offering support and guidance to parents on how to deal with this might aid them in the decision-making process at home.

Besides the two main processes of change that were discussed above, the present study also revealed a broad spectrum of behavioural changes, both between and within families. For instance, many participants reported healthy changes in one domain (e.g., dietary behaviours) while they did not change or even mentioned unhealthy changes related to the other domain (e.g., PA). Furthermore, several barriers that limited the transfer of behavioural changes to the home context were observed. Parents who did not report major changes often deemed change unnecessary for their family. They were satisfied with their current behaviour or found it too difficult to change their habits (e.g., due to financial or logistic challenges or a lack of knowledge). These motivational, financial, and practical barriers have previously been described in other research on parental perceptions regarding children’s dietary and PA behaviours [29,30,31,32]. Globally, three key factors necessary for behavioural change initiated by parents could be identified: (1) awareness of one’s behaviour, (2) willingness to change, and (3) ability to change (including e.g., financial and practical abilities and parental food literacy). These three prerequisites for behavioural change have previously been discussed under various names in other behaviour change research (e.g., the behaviour change wheel and the I-Change Model) [33,34].

The differences in the extent of change within and between families can be linked to the transtheoretical model (TTM). This model identifies five different stages of behavioural change through which people progress. Based on their degree of motivational readiness for change, people move from precontemplation (no intention to change) through contemplation, preparation, action, and ultimately maintenance (sustained change and resistance to relapse) [35]. The present study revealed a large variation in the degree of motivational readiness for change across participants. For example, there were participants who did not deem change necessary, which can be linked to being in the precontemplation phase. These participants reported little to no behavioural changes in their home context. Other participants mentioned that they had already formed new habits following HPSF implementation, which corresponds to being in the action or maintenance phase. In general, most behavioural changes in the home context were reported by participants who expressed an intention to change and were therefore in the contemplation, preparation, action, or maintenance phase according to the TTM.

The present study’s findings seem to contradict the results of a previous quantitative study on HPSF’s two-year effectiveness in the home context, where no statistically significant healthy or unhealthy changes in children’s dietary and/or PA behaviours were found [13]. However, it is debatable whether subtle behavioural changes can adequately be measured through quantitative instruments alone. Since behavioural change is often hard to identify, comes about slowly, and consists of various aspects, qualitative instruments might be more suitable to disclose all changes that may occur. Furthermore, a combination of quantitative and qualitative instruments is likely to result in a more in-depth picture of the phenomenon under investigation, as in this way, both explorative and explanatory information can be obtained [36,37,38]. The quantitatively observed four-year effects of HPSF seem to further support this idea [39]. After four years, significant positive intervention effects were observed for several objective outcome measures (e.g., children’s BMI z-score and waist circumference), while significant effects on quantitative outcome measures collected via self-report questionnaires (e.g., dietary behaviours) remained mostly absent after four years [39]. In the present qualitative study, however, participants reported to have experienced various behavioural changes in the home context, and it appeared that the concepts to measure these changes were addressed differently in the interviews than in the previously used quantitative self-report questionnaires. Due to the complexity of behavioural changes, it is often difficult for people to be aware of the changes that might have occurred. The qualitative nature of the interviews might have facilitated this as opposed to the self-report questionnaires. These observations underpin the value of taking a mixed-methods approach to investigate all potential intervention effects that may occur.

Several strengths and limitations of the present study should be discussed. Most of the qualitative literature on behavioural change focusses on the effectiveness of lifestyle interventions as perceived by participants or discusses the theoretical basis of change. To the best of our knowledge, this is one of the first qualitative studies examining the process of behavioural change in the home context after the implementation of a school-based lifestyle intervention. The study was executed in a region characterised by a low SES and a high incidence of overweight and obesity, which increased the relevance of investigating ways to maximise HPSF’s impact. However, it is not known if and how the intervention would have led to behavioural changes in the home context in other regions (e.g., regions characterised by a higher SES), which calls for the need to perform comparable research in more diverse regions and populations. Another limitation of the present study is that it was impossible to achieve investigator triangulation due to only one researcher coding the data [40]. However, notes were kept to reflect on the researcher’s objectivity, the ambiguities during coding were discussed with colleagues, and the interpretation of the data was conducted in close consultation with two other researchers. In addition, the risk of participants having provided socially desirable answers should be mentioned. Some participants only realised changes had occurred in the home setting once they started reflecting in response to the interviewer’s questions. Respondents might have engaged in impression management in an effort to cover up their true attitudes and behaviours. The researchers tried to minimise this risk by stressing confidentiality and the fact that participants could not give any wrong answers, and by carefully formulating the questions during the interviews. Another limitation is that HPSF’s role with regard to the perceived behavioural changes was not always clear, as other factors (e.g., ageing of children) might have also played a role in the observed changes. Interview participants were not always aware of behavioural changes that had occurred, and they were often not able to identify reasons for these changes. In addition, participants often indicated that behavioural changes, especially with regard to PA behaviours, occurred because their child grew older. Conducting interviews in the control schools could therefore have shed some more light on the degree of correlation between HPSF and the observed behavioural changes.

## 5. Recommendations for Future Research

As quantitative data collection instruments might not be suitable to detect all changes that may occur following implementation of health-promoting interventions, future studies investigating the effects of these interventions in various settings should adopt a mixed-methods approach and also include qualitative instruments. Furthermore, more research on the ways to stimulate child-to-parent IGL and improve parental involvement in school-based health-promoting interventions such as HPSF (e.g., through information provision regarding the intervention’s contents, organising parent–child activities such as cooking workshops, or providing information on how to deal with the justification of unhealthy compensatory behaviours that might occur at home) could lead to increased intervention effects in the home setting.

## 6. Conclusions

The present study is one of the first to provide insight into the processes, facilitators, and barriers of the transfer of behavioural changes acquired at school to the home context. Based on the observations presented in this paper, it can be concluded that school-based lifestyle interventions can lead to both healthy and unhealthy behavioural changes at home by influencing both children and parents. Both child-to-adult intergenerational learning and parent-initiated changes play an important role in the transfer of health behaviours from school to home. Further stimulating these mechanisms (e.g., by increasing parental involvement and support through family cooking workshops, homework assignments, or better information provision regarding the intervention components) can therefore lead to an increased impact of school-based health-promoting interventions’ impact in the home setting.

## Figures and Tables

**Table 1:** Characteristics of interview participants.

	N	%
School	27	
*HPSF1*	16	59.3
*HPSF2*	11	40.7
Caregiver interviewed	27	
*Mother*	20	75.0
*Father*	2	7.1
*Both*	4	14.3
*Other* ^a^	1	3.6
Location of the interview	27	
*Home*	14	51.9
*School*	12	44.4
*University*	1	3.7
Voted in favour of HPSF implementation (% yes)	21	77.8
Participation in regular HPSF measurements (% yes)	21	77.8
Familiarity with traditional primary school system (% yes) ^b^	21	77.8
Number of children in the family	27	
*One*	2	7.4
*Two*	20	74.1
*Three*	4	14.8
*Four*	1	3.7
Sex children ^c^	48	
*Male*	22	45.8
*Female*	26	54.2
Study group children ^c,d^	48	
*Lower (1–2)*	9	18.8
*Middle (3–5)*	21	43.8
*Higher (6–8)*	18	37.5

^a^ Grandmother and mother. ^b^ This could be because their child(ren) were already enrolled in one of the full HPSFs before implementation of HPSF or because their child(ren) were enrolled in another primary school. ^c^ Interview participants were recruited through these children, who were enrolled in the full HPSFs. ^d^ In the Dutch primary school structure, children successively follow eight “groups”, starting in group one at the age of four years and typically proceeding to secondary school at the age of eleven or twelve years. Internationally, the first two groups are comparable to preschool, and the last six groups are comparable to grades one to six. Abbreviations: HPSF, Healthy Primary School of the Future.

## Data Availability

Data supporting the study’s findings were collected as part of the “Healthy Primary School of the Future” quasi-experimental study. All individual participant data that underlie the results reported in this article (text, tables, figures, and appendices) will be available after de-identification. Data will be available with suitable qualified researchers beginning nine months and ending ten years following article publication on the four-year effects on the primary outcome measure of the original study (i.e., BMI z-score), as registered in the ClinicalTrials.gov database NCT02800616. Data will only be shared with parties who provide a methodologically sound proposal or for individual participant data meta-analysis. Proposals should be directed to mth.hahnraths@maastrichtuniversity.nl. To gain access, data requestors will need to sign a data access agreement. Three years following article publication on the four-year effects on the primary outcome measure of the original study (i.e., BMI z-score), data will be available in our university data warehouse but without investigator support other than deposited metadata.

## Supplementary Materials

The following are available online at https://www.mdpi.com/article/10.3390/ijerph182212219/s1, Supplementary File S1: Interview Guide (translated from Dutch).
