# Supplementary material for: “Mummy, Can I Join a Sports Club?” A Qualitative Study on the Impact of Health-Promoting Schools on Health Behaviours in the Home Setting
Source: Int J Environ Res Public Health. 2021 Nov 21;18(22):12219. doi: 10.3390/ijerph182212219 (PMC8620085; doi:10.3390/ijerph182212219)
Supplement: Supplementary file 1 [file ijerph-18-12219-s001.zip › ijerph-1405331-supplementary.pdf]

Supplementary File S1. Interview Guide (translated from Dutch)

Opening
Introduction of the interviewer: Good morning/afternoon, my name is … I am affiliated with the Healthy Primary School of the Future as a research assistant at Maastricht University. I will conduct this interview with you.

General points before the start of the interview:
- First of all, thank you for participating in this interview.
- A quiet environment ensures I can conduct this interview optimally.
- It would be preferred if we can both switch of our phones, so that we will not be interrupted.
- I would like to know about your experiences with the Healthy Primary School of the Future. Please be honest about your experiences. I am interested in positive as well as negative experiences. There are no right or wrong answers. We also like to hear negative points.
- Is it okay if I record this interview?
- Some questions are very personal. You do not have to answer them if you are not comfortable answering them.
- Everything you say during this interview will remain anonymous and confidential. Your name or your children's names will never be linked to this interview.
- What does your family situation look like? How many children attend the HPSF and are they participating in the research?

Introduction of HPSF
- What was your opinion about HPSF when it was introduced?
- A parental vote regarding the implementation of HPSF took place at your child's primary school. Can you still remember what your vote was and why?
- What were your children's opinions regarding HPSF when it was introduced?
- Did you take your children's opinions in consideration when voting?

Current situation
- What information did/do you receive about HPSF?
- Are you actively involved in HPSF? E.g., helping during the lunches, participating in parent feedback groups etc.
- How important is a healthy lifestyle for you and your family?
- What is your current opinion regarding HPSF? Did your opinion change over time?
- What are your children's current opinions regarding HPSF?
- Do your children talk about HPSF at home? Do they talk predominately about the positive or negative aspects of HPSF? Was there a change in the last few years in the way your children talk about HPSF?
- What are in your opinion positive aspects of HPSF and why?
- What are in your opinion negative aspects of HPSF and why?
- Do you notice an effect of HPSF on your children, yourself and/or the rest of the family (in general)?

Dietary behaviour
- Does HPSF have an influence on your family's dietary behaviour? If so, in what way (both positively and negatively)?
- What do your children tell you about the food/lunches at school?
- Did the dietary behaviour of your children change since the start of HPSF, both at school and in the home environment?
    - Ask specifically about breakfast, lunch, dinner, snacks, and drinks

- Do your children eat or drink different products since the start of HPSF? Who introduced these new products?
- Did the dietary behaviour of the rest of the family (siblings/parents/grandparents) change since the start of HPSF?
    - Ask specifically about breakfast, lunch, dinner, snacks, and drinks
    - Do siblings affect each other's dietary behaviour? Both positively and/or negatively.
- How do you lunch during the weekend (since the children lunch 4-5 days a week at school)?
- Do you see unhealthy/compensatory behaviours, e.g., more snacks or soft drinks at home?
    - Who initiates this behaviour? Do the children ask for more snacks or do the parents initiate the unhealthy/compensatory behaviours?
- Did your opinion about nutrition/food/drinks/diet change since the start of HPSF?
- Are there any dietary restrictions in your family? E.g., allergies, religious restrictions, medical restrictions

Physical activity

- Does HPSF have an influence on your family's physical activity behaviour? If so, in what way (both positively and negatively)?
- Is there a change in your children's physical activity behaviour since the start of HPSF?
- Is there a change in the physical activity behaviour of the rest of the family (siblings/parents/grandparents) since the start of HPSF?
- Did your opinion about physical activity change since the start of HPSF?

Other effects

- Do you notice an effect of HPSF on the overall well-being of your children?
- Do you notice other effects of HPSF on your children, yourself and/or your family?
- What is your opinion about screen time (e.g., TV, computer games, tablets etc.)?
- Are there any changes in dietary and/or physical activity behaviours you would like to see in your family, your children and/or yourself?
- What do you think about any possible long-term effects of HPSF, for example in 3-4 years, or when your children go to high school?

Concluding

- Do you have tips for other schools wanting to implement HPSF?
- Do you have tips to increase the effects of HPSF?
- Did we miss any topics you wanted to discuss?
